# Exploring the Potential Impact of GLP-1 Receptor Agonists on Substance Use, Compulsive Behavior, and Libido: Insights from Social Media Using a Mixed-Methods Approach

**DOI:** 10.3390/brainsci14060617

**Published:** 2024-06-20

**Authors:** Davide Arillotta, Giuseppe Floresta, G. Duccio Papanti Pelletier, Amira Guirguis, John Martin Corkery, Giovanni Martinotti, Fabrizio Schifano

**Affiliations:** 1 Department of Neurosciences, Psychology, Drug Research and Child Health, Section of Pharmacology and Toxicology, University of Florence, 50121 Florence, Italy; 2 Department of Drug and Health Sciences, University of Catania, 95124 Catania, Italy; giuseppe.floresta@unict.it; 3 Tolmezzo Community Mental Health Centre, ASUFC Mental Health Department, Via Giuliano Bonanni, 2, 33028 Tolmezzo, Italy; gducciopapantip@gmail.com; 4 Pharmacy, Faculty of Medicine, Health and Life Science, Swansea University, Singleton Campus, Swansea SA2 8PP, Wales, UK; amira.guirguis@swansea.ac.uk; 5 Psychopharmacology, Drug Misuse, and Novel Psychoactive Substances Research Unit, University of Hertfordshire, College Lane Campus, Hatfield AL10 9AB, UK; j.corkery@herts.ac.uk (J.M.C.); f.schifano@herts.ac.uk (F.S.); 6 Department of Neurosciences, Imaging and Clinical Sciences, University of Chieti-Pescara, 66100 Chieti, Italy; giovanni.martinotti@gmail.com; 7 Center for Advanced Studies and Technology (CAST), Institute of Advanced Biomedical Technology (ITAB), University of Chieti-Pescara, Via dei Vestini 21, 66100 Chieti, Italy

**Keywords:** GLP-1 receptor agonists, semaglutide, mental health, craving, substance, addiction, food noise, shopping, sex, social media

## Abstract

Glucagon-like peptide-1 (GLP-1) is involved in a range of central and peripheral pathways related to appetitive behavior. Hence, this study explored the effects of glucagon-like peptide-1 receptor agonists (GLP-1 RAs) on substance and behavioral addictions, including alcohol, caffeine, nicotine, cannabis, psychostimulants, compulsive shopping, and sex drive/libido. Data were collected from various social platforms. Keywords related to GLP-1 RAs and substance/behavioral addiction were used to extract relevant comments. The study employed a mixed-methods approach to analyze online discussions posted from December 2019 to June 2023 and collected using a specialized web application. Reddit entries were the focus here due to limited data from other platforms, such as TikTok and YouTube. A total of 5859 threads and related comments were extracted from six subreddits, which included threads about GLP-1 RAs drugs and associated brand names. To obtain relevant posts, keywords related to potential substance use and compulsive behavior were selected. Further analysis involved two main steps: (1) manually coding posts based on users’ references to the potential impact of GLP-1 RAs on substance use and non-substance habits, excluding irrelevant or unclear comments; (2) performing a thematic analysis on the dataset of keywords, using AI-assisted techniques followed by the manual revision of the generated themes. Second, a thematic analysis was performed on the keyword-related dataset, using AI-assisted techniques followed by the manual revision of the generated themes. In total, 29.75% of alcohol-related; 22.22% of caffeine-related; and 23.08% of nicotine-related comments clearly stated a cessation of the intake of these substances following the start of GLP-1 RAs prescription. Conversely, mixed results were found for cannabis intake, and only limited, anecdotal data were made available for cocaine, entactogens, and dissociative drugs’ misuse. Regarding behavioral addictions, 21.35% of comments reported a compulsive shopping interruption, whilst the sexual drive/libido elements reportedly increased in several users. The current mixed-methods approach appeared to be a useful tool in gaining insight into complex topics such as the effects of GLP-1 RAs on substance and non-substance addiction-related disorders; some GLP-1 RA-related mental health benefits could also be inferred from here. Overall, it appeared that GLP-1 RAs may show the potential to target both substance craving and maladaptive/addictive behaviors, although further empirical research is needed.

## 1. Introduction

Glucagon-like peptide-1 (GLP-1) is a multifaceted incretin hormone that plays a crucial role in glucose homeostasis and metabolic regulation. Its primary function is to stimulate insulin secretion in a glucose-dependent manner, which helps to reduce blood glucose levels. This action is achieved through the activation of GLP-1 receptors (GLP-1 Rs) on pancreatic beta cells, leading to increased insulin synthesis and secretion. This incretin activity has resulted in the approval of GLP-1 receptor agonists (GLP-1 RAs) for the management of type II diabetes [1]. Notably, GLP-1 is also involved in various independent central and peripheral pathways related to appetite regulation due to its anorexiant properties. To date, given their proven efficacy in producing weight loss, two GLP-1 RAs (e.g., liraglutide and semaglutide) are also being prescribed with this purpose [1,2] GLP-1 RAs such as semaglutide have shown a potential and significant influence in reshaping bodies, hence impacting the medical practice, food economy, and culture [3,4,5]. Apart from their social and economic impacts, GLP-1 RAs’ use goes beyond their conventional application in treating diabetes and obesity, potentially involving addictive and compulsive behaviors [6]. Substantial preclinical evidence in rodents and non-human primates has shown that GLP-1 RAs can modulate compulsive and reward-related behaviors in both the seeking and consumption aspects relating to various commonly abused substances (e.g., alcohol, nicotine, opioids and cocaine) [6,7]. GLP-1 RAs, at the central nervous system level, can impact the release of dopamine in the nucleus accumbens [6], thus modulating the rewarding effects of substance abuse, influencing drug-seeking behavior, diminishing responsiveness to drug cues, and probably alleviating the withdrawal symptoms’ experience [8].

The biological effects of GLP-1, mimicked by GLP-1 RAs (e.g., lixisenatide, liraglutide, exenatide, dulaglutide, and semaglutide), largely depend on the activation of GLP-1 Rs. GLP-1 Rs are expressed in multiple organs and tissues such as the pancreas, kidney, lung, immune system, stomach, bowel, cardiovascular system, and neurons within the enteric, central, and peripheral nervous systems [9]. Acute and chronic GLP-1 Rs’ activation triggers a complex network of intracellular signalling pathways (involving, e.g., cyclic adenosine monophosphate (cAMP), protein kinase A (PKA), protein kinase C (PKC), and calcium). These specific signalling patterns depend on the index GLP-1 RA; the target tissue; and the cellular context [9,10]. In the central nervous system (CNS), GLP-1 Rs are expressed in the cerebral cortex, hypothalamus, hippocampus, thalamus, substantia nigra, circumventricular organ, cerebellum, and brainstem nucleus, with GLP-1 Rs’ gene expression pattern evident in rodents, non-human primates, and humans [10]. Acting in these CNS structures, GLP-1 RAs exert a wide range of effects, including those on satiety, energy homeostasis, and neurogenesis [10]. Based on their ability to modulate the mesolimbic reward system [11] and food intake, different authors have hypothesized that GLP-1 RAs might regulate drug-motivated behaviors and drug-reward mechanisms [7,12]. Moreover, according to recent studies [10,13], some GLP-1 RAs, due to their extended half-lives, exhibited the capability of crossing the blood–brain barrier and exerting effects in the brain that could differ from those of endogenous GLP-1. GLP-1 RAs may potentially be involved in further neurotransmitter (e.g., serotonin; dopamine) activities [14]. It has been suggested that food intake and substance use disorder share common neurobiological mechanisms [15]. The reward system is a complex network, presenting interactions between the opioid, dopamine, endocannabinoid, and serotonin systems; indeed, food may act as a stimulus to feeding due to its rewarding nature [16].

According to the potential GLP-1 RAs’ central and dopamine signalling mechanisms of action, the likelihood of GLP-1 RAs being used clinically for addictions is promising [6,8]. Nevertheless, a range of issues limiting the use of GLP-1RAs in patients with addictive disorders can be identified, including possible individual variations associated with, e.g., gender and genetic differences; unproven efficacy and safety; unclear mechanisms of action; the risk of pharmacological interactions and side effects; and the presence of psychiatric comorbidities in substance use disorder subjects.

Ongoing clinical trials and real-world analysis [17,18] are being carried out to assess these encouraging preclinical findings’ translatability to humans. Further research, however, needed to understand the real impact of GLP-1 RAs on addictions; identify patient subgroups that may benefit from them; and evaluate their long-term efficacy and safety, also in comparison with traditional addiction treatments. Current human research is primarily concentrating on alcohol and nicotine abuse with co-occurring metabolic disease; conversely, there is only limited/controversial knowledge relating to both preclinical and real-world scenarios regarding other substances of abuse such as cocaine [11], opioids [19], entactogens, psychedelics, dissociative drugs, and non-substance/behavioral addictions.

Understanding the impact of GLP-1 RAs on mental health outcomes is complex, due to both the subjects’ individual differences and potential biases in data analysis [20,21,22]. Moreover, both obesity and type 2 diabetes are multifactorial and complex diseases, and their exact inter-relationship with mental health and addiction are not fully understood, including the psychopathological issues linked to food-related emotional and behavioral aspects [1,21]. Other unclear aspects may involve the interconnection between GLP-1 receptors and a range of cognitive-, immunological-, and inflammation-related issues [23,24,25].

Drug repurposing is a useful strategy for identifying new uses for approved or investigational drugs and extending their range of therapeutic use [26]. The post-marketing surveillance of adverse drug reactions, using both social media listening and international agencies’ pharmacovigilance databases, is another useful approach for highlighting potential critical issues related to the use of medicines [27,28]. Despite its limitations, social platforms’/forums’ analysis is a feasible approach as well for exploring the multifaceted aspects of drug use/misuse patterns [29]. These methods, initially used mainly in marketing and social sciences, have recently attracted increasing levels of interest in the scientific community, given both the obvious difficulties/limitations in carrying out clinical trials and the partial inadequacy of data that can be collected in clinics. Hence, “netnography”, when combined with content analysis and qualitative and quantitative analysis [21,30], may offer intriguing opportunities to better understand the patterns of substance use. When used with extreme care and constant human control, the vast amount of web data have also opened the door to the use of machine learning and generative artificial intelligence (AI) as interpretive and analytical support tools [31,32].

Drawing our previous work [21], and applying the web-based qualitative and quantitative methods, we aimed at exploring the potential effects of GLP-1 RAs on possible modifications relating to both drug/alcohol abuse intake and behavioral addictions [18,33].

## 2. Materials and Methods

Employing an unobtrusive, mixed-methods, qualitative, quantitative, and netnographic approach [34,35,36], data were collected from Reddit, TikTok, and YouTube [14,37,38]; their related content was freely available from the open web. These platforms were chosen for their global reach, large user base, and significant impact on online interactions and content sharing. Data collection was carried out in two phases: the first one on 28 May 2023, whilst an update was obtained on 13 June 2023. Both phases were carried out with the help of a specialized web application [39]. Some 5859 threads and 12,136 related comments were extracted from six subreddits. The web application retrospectively collected all entries made since the inception of the different subreddits examined here, which were created over a timeframe spanning from December 2019 to November 2022. Most social media users’ comments, however, were made during the Spring of 2023. Due to paucity of both TikTok- and YouTube-related data, only the collected Reddit posts were analyzed here. Reddit is a web-based platform that organizes topics into distinct forums (subreddits), where each discussion is considered a thread. This popular platform hosts a great variety of topics, which are typically discussed by a large plethora of users (Redditors) [40,41]. Reddit entries, being both anonymous and voluntary, have become a popular source for social listening studies [42,43]. 

Information of interest was searched whilst focusing on a range of medications, including Ozempic, semaglutide, tirzepatide, Mounjaro, Wegovy, and Rybelsus. In association with this, the following subreddits were analyzed: r/Ozempic, r/OzempicForWeightLoss, r/Semaglutide, r/Tirzepatide, r/WegovyWeightLoss, and r/Mounjaro). Other keywords were also included in the search (e.g., dulaglutide, liraglutide, albiglutide, exenatide, lixisenatide, Trulicity, Victoza, and Saxenda), but only few/very few related comments were identified, and hence, they were considered irrelevant for the current analysis. The collected raw data were imported into Word and Microsoft Excel spreadsheets (Microsoft Office Professional 2021, Version 2308 Build 16731.20170). A range of selected keywords of interest (e.g., alcohol; caffeine; nicotine; cannabis; cocaine; compulsive shopping; libido; see Table 1 and Table 2 for the complete list) were then searched within the archived whole set of comments. The resulting comments containing the same keyword(s) were then grouped together and analyzed both manually and using a specialized software (Numerous.ai). A multiple-step strategy was then adopted, as follows: 

(A) Quali-quantitative analysis. After having been classified according to GLP1-RAs’ impact on substance consumption (e.g., increased, reduced, stopped), each comment was manually coded. Trivial/not relevant comments were excluded, and the total number of coded comments are shown in Table 1 and Table 2. 

(B) Thematic analysis. Comments were sampled in groups (See Table 1 and Table 2). The resulting groups, containing the same keyword(s), were then analyzed using a specialized software (Numerous.ai). The whole set of themes and biases relating to each keyword generated by Numerous.ai was then manually revised. The following prompt was used to analyze each group of comments [= ai(“I want you to act as an expert in qualitative content analysis and analyze these posts for me. identify all the themes and then present them in bullet points. please also consider any potential biases or contextual factors that may impact your analysis”)]. Each set of themes was further analyzed using ChatGPT 3.5 using the following prompt: “could you identify the 5 most frequent themes/biases among these?”. For data assessment and interpretation, a phenomenological qualitative media analysis [44] was undertaken as well. In line with similar studies [45,46], for each keyword selected, here, the most common resulting themes were then analyzed manually. In this way, a list of main themes was obtained (Table 3; Supplementary Material Table S1). Two independent researchers (DA and GF), with different backgrounds in qualitative research, at times involving the supervision of a senior researcher/author (FS), independently analyzed all the posts and related comments. After the appropriate discussion and contextualization of the resulting comments, issues relating to disagreements on coding were resolved. The comments were analyzed in their entirety, without cleaning them and whatever their length, thus maintaining their originality. No chatbot training/data pre-processing strategies [47,48,49] were performed. According to the unobtrusive and naturalistic methods of conducting netnographic studies [34,35,36], no posts or other contributions to private/public forum discussions were made. In line with previous studies [36,50,51], full anonymity was guaranteed, and no usernames or references to the Redditors were collected, used, or analyzed. 

## 3. Results

The current dataset consisted of a large range of comments pulled from various platforms (Reddit, TikTok, and YouTube). The keywords “Ozempic”, “semaglutide”, “tirzepatide”, “Mounjaro”, “Wegovy”, and “Rybelsus” attracted the most attention and were analyzed here, consistent with previous data from our group [21]. Reddit posts (e.g., individual elements within a thread) yielded 12,136 comments, YouTube videos yielded 14,515, and TikTok videos yielded 17,059, respectively. Further 5859 Reddit threads were also identified as a potential source for identifying additional comments. Reddit threads were extracted from the following subreddits: r/Ozempic; r/OzempicForWeightLoss; r/Semaglutide; r/Tirzepatide; r/WegovyWeightLoss, and r/Mounjaro. Conversely, Table 1 and Table 2 report the number of matches from all Reddit comments (n = 1020) for the searched keywords (e.g., alcohol; caffeine; nicotine; cannabis; cocaine; compulsive shopping; libido). The keywords were further categorized in topics. A qualitative analysis was then performed using an assisted AI methodology (see above), which led to the identification of the five most common themes for the whole keyword-related dataset (Table 3) and for each keyword (Supplementary Material Table S1). Supplementary Material Table S1 shows a qualitative analysis of substance and non-substance abuse-related patterns relating to weight loss/GLP-1 RAs treatments and examples of relevant posts. 

As can be seen in Supplementary Material Table S1, a wide range of topics were covered, highlighting the multifaceted and interconnected relationship between addiction, craving, and managing weight. When examined separately, these topics showed a tendency for overlapping findings. Consequently, certain areas were examined more closely, and pertinent, exemplifying posts were presented in Supplementary Material Table S1 to further elucidate these connections.

### 3.1. The Complex Interrelation between Weight Loss Drugs and Substance-Related Use/Craving

Alcohol. Users generically showed levels of optimism regarding GLP-1 RAs’ potential benefits in alcohol-related issues (e.g., reduced cravings and changes in alcohol tolerance); they also highlighted these molecules’ beneficial effects in improving overall well-being, e.g., eating more proteins, high-fibre fruits, and vegetables; avoiding certain foods such as deep-fried foods and heavy cream sauces; and giving preference to alcohol-free beers. This shifting in consumption habits was at times reportedly related to modifications in taste preferences. On the other hand, others complained of their frustration associated with the impossibility of enjoying even low doses of alcoholic drinks anymore. The consumption of different types of alcohol (e.g., wine vs. beer) did not show clear differences, although their different composition and consumption patterns could explain those minor differences being highlighted. Most reporters seemed to be worried of GLP-1 RAs medications’ access (e.g., financial barriers, medications supply) and complained about the insurance coverage limitations. Conversely, a few users reported an increase in alcohol consumption. Whilst the veracity of these contributions, which were small in number, may not be confirmed here, it is possible that such users showed a provocative attitude due to personological issues. Overall, some 29.75% of alcohol-related comments clearly stated a cessation of the intake of these substances following the start of the GLP-1 RAs prescription.

Caffeine. Certain users acknowledged the role of caffeine as an effective appetite suppressant, agreeing on its integration into weight loss strategies to boost metabolism and control food cravings. Other reporters emphasized that caffeine may interfere with carbohydrate absorption and glucose release modulation, with the ingestion of limited dosages having been widely accepted. Positive outcomes from maintaining caffeine consumption (e.g., improved alertness and productivity) were also reported. Further, some users noted improvements in sleep quality, reduced anxiety levels, and a decreased desire for caffeinated beverages, possibly influenced by the reward/satiation/hedonic modulation associated with GLP-1 RAs’ intake. The well-known potential withdrawal symptoms and addictive nature of caffeine were discussed, along with individual variability in caffeine tolerance. Coffee’s impact on GLP-1 RAs’ pharmacokinetics was discussed here, along with factors like acidity, gastrointestinal motility, individual differences, and consumption habits; differences in responses were linked to inappropriate time intervals between caffeine and medication intake. Overall, some 22.22% of caffeine-related comments reported a cessation of the intake of coffee beverages in association with GLP-1 RAs.

Nicotine. The users’ experiences with smoking cessation ranged from the intake of occasional cigarettes to quitting altogether; some noted a significant reduction in cravings and others mentioned overall changes in pleasure while using GLP-1 RAs. Some users experienced immediate effects, whilst others noted a gradual shift in their smoking behavior. An awareness of psychosocial factors, stress, mental health, and coping mechanisms influencing smoking cessation was evident among users, with posts emphasizing these interconnected influences on individual choices. Overall, some 23.08% of nicotine-related comments reported a cessation of the intake of tis alkaloid in association with GLP-1 RAs.

Cannabis-derived products. Cannabis-related discussions focused on its impact on appetite modification, with users noting changes in hunger levels, including increased levels of appetite, mainly attributed to “munchies” from cannabis. Nausea relief through cannabis use was also discussed, particularly in alleviating nausea caused by medications like Ozempic Interestingly, users considered the contrast between THC-related relief from Ozempic-associated nausea and THC-associated increased appetite levels. 

Cocaine and remaining stimulants. Cocaine and amphetamine-like medications were frequently cited as potent appetite suppressants; users commented on their ability to both eliminate hunger and impact eating habits and body weight. The discussions around stimulants displayed sarcastic and provocative tones; a range of comments relating to Ozempic revealed a level of cynicism towards the medical industry. 

### 3.2. Weight Loss Medication Intake and Sex Drive/Libido

Sexual drive. The experiences varied, with some reporting an increase in libido and others being curious about possible links between medications and changes in sexual desire. Some users reported an increase in libido after losing weight and an overall improvement in their sexual arousal. Where sexual improvements were reported, various factors were reportedly involved, including a weight loss-related positive effect on perceived body image; the role of insulin control in influencing the sexual hormones; levels; and the overall impact on mental health. Possible disease- and age-related components of libido changes were proposed by some to explain the sexual drive modification.

### 3.3. Weight Loss Drugs and Behavioral Addictions (Unrelated to Food)

Following GLP-1 RAs’ intake, a number of users reported a decrease in both impulsive and compulsive shopping behaviors, shifting to more controlled and planned shopping habits. Changes in shopping frequency and spending patterns were noted, with users becoming more aware of the consequences of impulsive buying and prioritizing financial responsibilities. Conversely, others did not experience the same positive outcomes. Overall, 21.35% of comments reported a GLP-1 RA-related compulsive shopping interruption, whilst the sexual drive/libido elements reportedly increased in several users.

## 4. Discussion

To the best of our knowledge, this is the first paper aiming at exploring, with a mixed-methods approach, the potential impact of GLP-1 RAs on substance-related and behavioral addictions. The focus was here on both legal (alcohol, caffeine, nicotine) and recreational (cannabinoids, psychostimulants, and other substances) drugs. Data relating to possible changes in compulsive shopping activities and sexual drive were provided here as well. 

Social media analysis has the potential to provide valuable real-time data for researchers, clinicians, and stakeholders across various fields [36]. The usability, accessibility, and immediacy of social media platforms create a unique opportunity for individuals to share experiences, connect with others, and access resources. When applied to drug abuse and behavioral research, social media analysis could potentially contribute to expanding clinicians’ knowledge in diagnosing and treating potential addiction issues. For example, analyzing social media posts and online activities can help in identifying potential triggers and patterns of substance misuse and addictive behavior [52]. Furthermore, in the context of precision medicine, examining social media behavior can aid in personalizing treatment approaches by enabling healthcare professionals to both better understand the individual needs and develop targeted strategies within formal healthcare settings. Moreover, the netnographic approach in medicine can be considered cost-effective due to its rapid and efficient data collection; access to a wide range of participants; in-depth analysis of online interactions; and potential for real-time data analysis.

Alcohol. Consistent with previous data, a significant impact of GLP-1 RAs on alcohol craving and intake levels was observed here [18,53]. Similarly to what was observed with remaining substances commented on here, this reduction in alcohol intake levels could be better understood whilst considering three factors: (a) people being prescribed with GLP-1 RAs stopped/reduced their consumption as part of a lifestyle change; (b) subjects who have done the same as a possible direct consequence of GLP-1 RAs; (c) people who no longer enjoyed drinking alcohol because they experienced pronounced side effects (e.g., malaise; nausea) in association with the GLP-1 RAs’ intake.

Indeed, lifestyle changes may well be associated with reduced levels of psychotropic substances consumption [54]. Furthermore, both the beneficial effect of GLP-1 RAs on psychopathological aspects (e.g., improved mood) [21,55] and the significant weight loss-related improvement in one’s appearance [22,56,57,58] could also be hypothesized as being significant factors contributing to reduced drug consumption levels. Previous research has also suggested both direct and indirect effects of GLP-1 RAs on craving, pleasure circuits, and extended networks of substance abuse-related receptor mechanisms [6,59]. Finally, the direct adverse effects of GLP-1 RAs in the context of alcohol abuse are likely to be mainly of gastroenterological relevance [60], hence acting as anti-addictive/aversive stimuli. Moreover, even if GLP-1 RAs might temporarily help in preventing the occurrence of alcohol-related incidents during the early stages of therapy [60], they may not be suitable for all individuals with alcohol use disorder because of these molecules’ potential adverse effects (e.g., malaise and risk of pancreatitis), particularly in individuals with a low body weight or pre-existing pancreatic conditions [6].

Caffeine. Little is known about the actual effects of caffeine whilst on GLP-1 RAs medications. Coffee drinks should have a slight effect on both GLP-1 R and GLP-1 RAs’ pharmacokinetics, due to various factors such as the presence of acids and other compounds (e.g., chlorogenic acid and polyphenols) [61,62]; their effects on gastrointestinal motility; potential individual differences; and differing individuals’ consumption styles (e.g., large, long-lasting, and repeated beverage sessions; an adjunct of sugar/fatty ingredients in the drink). Caffeine intake is also related to weight loss maintenance [63]. On the other hand, coffee contains beneficial nutritional elements for carbohydrate absorption and glucose release modulation, and its consumption, in limited quantities, is widely accepted. 

Nicotine and cannabis-derived products. Preliminary studies suggest that GLP-1 RAs have the potential to reduce the nicotine reward, decrease the nicotine intake, and potentially increase smoking abstinence rates [64,65]. Interestingly, molecules such as dulaglutide have demonstrated benefits in preventing post-cessation weight gain, suggesting that these medications could help in managing the metabolic issues that often accompany quitting smoking [66].

In relation to cannabis, recent findings showed preliminary evidence of the potential benefit of semaglutide in cannabis use disorder in distinct populations with obesity and type 2 diabetes [67]. Current data may confirm the occurrence of a possible reduction in cannabis intake levels in association with semaglutide.

Cocaine and remaining stimulants. Preclinical findings showed promising results supporting GLP-1 RAs as potential cocaine use disorder medications [6,68]. Among the underlying mechanisms, some authors hypothesized that GLP-1 RAs can attenuate the cocaine reward by both regulating some neurotransmitter release (e.g., dopamine, GABA, glutamate) and reducing inflammation [68]. To date, however, there is no clear evidence that GLP-1 RAs have an effect on changes in stimulant misusing levels in a clinical setting. Indeed, some authors highlighted the relationships between binge eating and stimulant abuse [69], whilst others aimed at clarifying the role of dopamine in the binge eating context [70]. In humans, GLP-1 RAs may not have a significant effect on pervasive and typical stimulant behavioral sensitisation [71]. Rather than substantially affecting dopamine reuptake, one could then argue that these molecules may be able to modulate it only partially or indirectly, and this is consistent with current findings. Similar considerations could be made for the amphetamine-type substances, which have indeed been discussed as popular weight loss agents.

Entactogens, psychedelics, dissociative drugs, and other abusing drugs’ intake. No posts commenting on opioid misuse were identified here, despite a few preclinical findings that reported the possible influence of GLP-1 RAs on opioids intake [1]. Conversely, a few comments reported slightly enhanced/extended effects, with a delayed onset, of MDMA, arguably due to the GLP1-RA-associated delayed gastric emptying. Similar findings were reported in relation to both psychedelic mushrooms and ketamine [6,8,15,16]. It is of interest that massive levels of acute MDMA intoxication have been associated with hypoglycaemic states [72]. 

Sexual drive. Little is known about GLP-1 RAs’ potential effects on libido and sexual behavior [73]. Whilst the GLP-1 receptor activation may reduce the levels of sexual interactions in mice [74], improvements in libido and sexual function have been observed in males using GLP-1 RAs [75]. Indeed, long-acting GLP-1 RAs may present the potential to boost erectile function in males with type 2 diabetes mellitus [76]. Current clinical evidence shows promising results in both female and male fertility levels, due to weight loss and/or direct actions on the reproductive system [77].

General considerations regarding reward-related/compulsive behaviors (e.g., food eating, shopping, gaming, internet addiction). GLP-1 RAs’ effects seemed to extend not only to food satiety but also to drug craving and various other behavioral disorders (e.g., compulsive shopping), involving the perceived pleasure of substance use and the aversion to excessive patterns in a transversal way. The following questions, therefore, arise: (a) how much/many impulsivity/compulsive mechanisms is/are involved; (b) is there is an influence of GLP-1 RAs on emotional aspects/stress; and (c) what is the specific role of the different GLP1-RAs in modifying behaviors, habits, and chemical dependencies. As we refer to a dual diagnosis (substance use disorder plus a psychiatric disorder), a triple diagnosis (metabolic disease plus substance use disorder plus psychiatric disorder) clinical cluster may be emerging as well. Indeed, individuals should be considered as a composite unit, and it is more appropriate to stratify for different risk factors, subpopulations, and biotypes [78]. 

Shifting research away from addiction per se and broadening the focus on the underlying psychopathological issues, we should aim at understanding why the same underlying conditions manifest in different ways in different individuals. A more integrated perspective should include contributions from eating disorders; inflammation [79,80]; food addiction; and impulsivity [81,82] studies. All these issues may shed some light on the interpretation of some of the current results. In this context, whilst the ability of GLP-1 RAs agonists to cross the blood–brain barrier is still controversial [12,83,84], the potential importance of its integrity, e.g., in terms of gut–microbiome–brain axis crosstalk, dysbiosis in obesity, alcoholism, substance use disorders, and systemic inflammations [85,86,87], may be of particular interest. 

Current data suggest that the benefits of GLP-1 RAs may be evident from their first ingestions and appear to last as long as GLP-1 RAs are being administered. We found here, however, only limited information on the effects of GLP-1 RA tapering/discontinuation. One could argue, however, that GLP-1 RAs’ duration of effects may be transient [88,89], although this clearly requires further investigation. 

Regarding alcohol, this is a substance whose consumption, albeit at moderate dosages, is quite prevalent in the general population; hence, GLP-1 RA-related changes may revolve around a reduction in alcohol intake levels. Similarly, the remaining behaviors analyzed here are likely to be merely modulated, mainly towards a reduction in reward-/compulsive-related behavior, as shown in preclinical findings [9,12]. Overall, users preferred to continue taking GLP-1 RAs as prescribed to maintain the metabolic effects; overall, they did not appear to be concerned about any possible compulsive and reward-related behavioral changes [90,91].

Some concerns emerged here in relation to the non-prescribed intake of further medications in combination with GLP1-RAs, e.g., with the appetite suppressant tesofensine, or with other molecules taken with the intention to manage GLP-1 RAs’ side effects/consequences, such as skin loss. Particular attention should be paid to certain populations at risk of GLP1-RAs abuse, such as pregnant/lactating women [92,93]; underweight subjects taking psychostimulants [69]; eating-disordered individuals [94]; the elderly [95]; and people with reduced muscle mass levels [96]. 

Finally, consistent with previous findings [21,97], GLP-1 RAs probably helped to cut the “food noise” (e.g., food-related brain ruminations) being experienced by subjects. Interestingly, treatment with semaglutide is also related to reductions in substance use or compulsive behaviors other than eating [97]. Alterations in dopaminergic pathways in the preclinical model and in human brains both contribute to substance misuse and play a role in compulsive behaviors [98,99], leading to the hypothesis that GLP-1 RAs can act on several factors influencing different maladaptive behaviors, manifesting cross-cutting effects on behavioral and non-behavioral addictions [59]. The overlap between food and substance cravings at a neural level shows the intricate connection between brain reward pathways. GLP-1 RAs could offer a new way to address this overlap by modulating these pathways, potentially reducing the reinforcing effects of both food and substances. By targeting GLP-1 receptors in the brain, these drugs can affect the reward system and areas linked to impulse control and decision-making, suggesting a “dual action” on appetite regulation and addictive behaviors. Overall, targeting “noise” associated with cravings for food and substances with GLP-1 RAs could offer new avenues for managing addictive behaviors and promoting healthier habits.

Limitations. The limitations of this study include biases typically relating to social media-based studies, such as the use of self-reported data, which may be associated with possible reporting inaccuracies and omissions. The current data related only to what was anecdotally reported by social media users, whose social, economic, and demographic attributes may not accurately reflect the whole society. Further, as previously highlighted [100], it is also possible that the impact of fake accounts somehow affected our data. Additionally, social media user-related biases may underscore the importance of cautious interpretation and the integration of alternative methodologies in social media studies [101]. Researcher bias, linguistic heterogeneity, and self-presentation tendencies may also affect the rigor of the data. The use of English as the sole language for analysis and the choice of keywords may have introduced a selection bias as well, potentially excluding relevant discussions. Demographic differences between platforms and different users’ characteristics may affect the generalizability of the study. The current analysis did not examine the differences between GLP-1 RAs’ routes of administration nor compared individual molecules, e.g., tirzepatide was less likely to be discussed by users than semaglutide. The use of AI and natural language processing for text analysis could have introduced further bias and error, and the potential lack of transparency still required constant human oversight. Finally, it was unclear whether the use of psychoactive substances by current internet users effectively constituted an unhealthy habit; a substance abuse/misuse; or an addiction condition. For these reasons, further clinical trials are needed.

Despite the potential limitations given by the reliance on self-reported statements and qualitative analysis, a wide range of findings and insights were extracted and discussed, thereby partially validating the limitations of the dataset. The long-term sustainability of these behavioral changes is an aspect that may require ongoing monitoring. 

## 5. Conclusions

GLP-1 RAs such as semaglutide and tirzepatide were suggested to possess levels of positive effects on reducing both substance- (e.g., alcohol; nicotine; and caffeine) and non-substance-related (e.g., compulsive shopping) behaviors. Conversely, the current findings were at times inconsistent/contradictory, with some comments indicating a persistence/recurrence of substance-related cravings over time. While the limited data collected in this study apparently suggest that GLP-1 RAs may potentially exhibit some degree of a positive effect on reducing both substance- and non-substance-related (e.g., compulsive shopping) behaviors, these findings should be interpreted with caution. The discussed mechanisms involving reward and satiation are speculative and require further investigation to establish causality. The potential effects on alcohol, nicotine, and caffeine are in line with some previous preliminary findings, but the actual effects on cannabis-derived products; psychostimulants such as cocaine; opioids; and other substances remain unclear. Conversely, intriguing novel insights came from aspects related to libido. The small sample size, the time-limited data collection, and the self-reported nature of the social media data used in this study may well limit the generalizability and reliability of the findings. Rigorous research, including well-designed clinical trials and the analysis of real-world data, is needed to substantiate these preliminary observations and accurately determine the therapeutic potential of GLP-1 RAs in treating substance use disorders and behavioral addictions. More robust epidemiological and clinical research is warranted before any definitive conclusions can be drawn. 

Overall, the advantages of netnographic strategies in investigating real-life experiences and perceptions concerning pharmaceutical interventions were emphasized here, while also acknowledging the limitations inherent in social media data. The intricate interplay between a wide range of factors, including body image, sexual health, individual characteristics, and the GLP-1 RAs’ effects, suggests the need for a holistic understanding of these interrelated issues. The complex nature of addiction, craving, and weight management necessitates further research to be carried out to better understand the mechanisms and long-term effects of GLP-1RAs. 

## Figures and Tables

**Table 1:** Quantitative analysis. Substance and non-substance addiction patterns.

***Substance addictions/craving for commonly used drugs (legal and not legal)***						
**Alcohol, alcoholism**						
Keywords	stopped	reduced	same	increased	other topics *	total
*alcohol*	123	60	31	2	169	385
*alcoholism*	0	0	0	0	17	17
*alcoholic*	7	3	0	0	25	35
	130 (29.75%)	63 (14.42%)	31 (7.09%)	2 (0.46%)	211 (48.28%)	437
**Other drinking habits: coffee drinks**						
Keywords	stopped	reduced	same	increased	other topics *	total
*caffeine*	10	3	4	0	16	33
*coffee*	38	26	72	0	47	183
	48 (22.22%)	29 (13.43%)	76 (35.19%)	0 (0.00%)	63 (29.17%)	216
**Tobacco**						
Keywords	stopped	reduced	same	increased	other topics *	total
*nicotine*	1	0	0	0	0	1
*cigarette*	2	1	0	0	9	12
	3 (23.08%)	1 (7.69%)	0 (0.00%)	0 (0.00%)	9 (69.23%)	13
**Cannabis-derived products (inhaled and edibles)**						
Keywords	stopped	reduced	same	increased	other topics *	total
*cannabis*	2	1	2	0	4	9
*weed*	0	1	1	0	15	17
*edibles*	9	9	9	9	9	45
*munchies*	1	2	6	0	10	19
	12 (13.33%)	13 (14.44%)	18 (20.00%)	9 (10.00%)	38 (42.22%)	90
**Vaping and smoking/inhaling habits (excluding psychostimulants such as cocaine and amphetamines): tobacco and cannabis-derived products**						
Keywords	stopped	reduced	same	increased	other topics *	total
*vaping*	1	2	1	0	2	6
*smoke*	3	4	11	0	22	40
*smoking*	18	1	6	1	44	70
	22 (18.97%)	7 (6.03%)	18 (15.52%)	1 (0.86%)	68 (58.62%)	116
***Sex drive and libido***						
Keywords	stopped	reduced	same	increased	other topics *	total
*sex*	0	1	0	5	27	33
*libido*	0	1	0	1	2	4
	0 (0.00%)	2 (5.41%)	0 (0.00%)	6 (16.22%)	29 (78.38%)	37
***Behavioral addictions (excluding food)***						
**Shopping**						
Keywords	stopped	reduced	same	increased	other topics *	total
*compulsive shopping*	2	0	0	0	1	3
*shopping*	19	6	14	2	45	86
	21 (23.60%)	6 (6.74%)	14 (15.73%)	2 (2.25%)	46 (51.69%)	89

*: here, posts with the selected keywords but falling outside a clear description of how consumption changed (e.g., trivial comments, generic questions, searching for reassurances, links, etc.) were included. A more conservative approach was adopted, and “stopped/reduced/same/increased” was coded only if clearly stated.

**Table 2:** Quantitative null analysis; substance and non-substance abuse-related patterns.

**Psychostimulants: cocaine and amphetamines**						
Keywords	stopped	reduced	same	increased	other topics *	total
*cocaine*	0	0	0	0	10	10
*amphetamine*	0	0	0	0	8	8
	0 (0.00%)	0 (0.00%)	0 (0.00%)	0 (0.00%)	18 (100%)	18
**Other substances (e.g., opioids such as fentanyl, heroin; painkillers; benzodiazepines; ketamine; empathogens such as MDMA; psychedelics such as magic mushrooms and psilocybin): none/not relevant**						
	stopped	reduced	same	increased	other topics *	total
*/*	0	0	0	0	0	0
	0 (0.00%)	0 (0.00%)	0 (0.00%)	0 (0.00%)	0 (0.00%)	0
**Gaming and internet addiction**						
Keywords	stopped	reduced	same	increased	other topics *	total
*gaming*	0	0	0	0	4	4
*internet addiction*	0	0	0	0	0	0
	0 (0.00%)	0 (0.00%)	0 (0.00%)	0 (0.00%)	4 (100%)	4
**Other behavioral addictions (e.g., gambling): none/not relevant**						
	stopped	reduced	same	increased	other topics *	total
*/*	0	0	0	0	0	0
	0 (0.00%)	0 (0.00%)	0 (0.00%)	0 (0.00%)	0 (0.00%)	0

*: here, posts with the selected keywords but falling outside a clear description of how consumption changed (e.g., trivial comments, generic questions, searching for reassurances, links, etc.) were included. A more conservative approach was adopted, and “stopped/reduced/same/increased” was coded only if clearly stated.

**Table 3:** The five most common themes overall.

Themes
*Medication effects and side effects*: this theme includes discussions about the effects of GLP-1RAs on habits, appetite, cravings, and weight loss. Potential side effects and health risks associated with these medications, such as digestive issues and long-term impacts on health, are also discussed here.
*Lifestyle changes and weight management*: users discuss the importance of making lifestyle changes alongside medication intake for effective weight management (e.g., incorporating healthier dietary habits, engaging in physical activity, and other lifestyle modifications for supporting weight loss efforts).
*Substance use and cravings reduction*: the users focus on the reduction in cravings for substances like alcohol, cigarettes, and sweets while on GLP-1RAs. Users also share their experiences with managing or quitting substance use.
*Individual experiences and responses to GLP-1RAs*: this theme includes a range of individual experiences with medications (e.g., both positive and negative outcomes). Users share personal stories of weight loss progress, side effects, and overall responses to treatment, providing insights into the varied experiences with these medications.
*Health risks, regulation, and societal perceptions*: discussions highlight users’ concerns about health risks associated with medications and substance use, as well as societal perceptions and regulatory inconsistencies (e.g., in the context of weight loss advertising and the pharmaceutical industry).

## Data Availability

The data that support the findings of this study are available from the corresponding author upon reasonable request due to potential ethical issues.

## Supplementary Materials

The following supporting information can be downloaded at: https://www.mdpi.com/article/10.3390/brainsci14060617/s1, Table S1: Qualitative analysis. Substance and non-substance addiction-related themes; examples of relevant posts; the five most common themes for each group of keywords.
