# Supplementary material for: Exploring the Potential Impact of GLP-1 Receptor Agonists on Substance Use, Compulsive Behavior, and Libido: Insights from Social Media Using a Mixed-Methods Approach
Source: Brain Sci. 2024 Jun 20;14(6):617. doi: 10.3390/brainsci14060617 (PMC11202225; doi:10.3390/brainsci14060617)
Supplement: Supplementary file 1 [file brainsci-14-00617-s001.zip › brainsci-3037025-supplementary.pdf]

Supplementary Material Table S1. Qualitative analysis. Substance and non-substance addiction-related themes; examples of relevant posts; the five most common themes for each group of keywords.

Themes

Substance addictions/cravings relating to the most typically misused drugs (legal and not legal)

Alcohol. Keywords: alcohol; alcoholism; alcoholic

- *Impact of medications:* many users generically discussed the effects of Ozempic and Mounjaro on their habits. Some users reported changes in taste preferences and a decrease in cravings for alcohol and other substances (such as sweets and carbohydrates).
- *Weight loss and lifestyle changes*: discussions included the effects of these medications on appetite and dietary habits. Users also highlighted the importance of making healthy lifestyle changes (for e.g., physical activity and dietary modifications, alongside the medication intake).
- *Side effects and health concerns*: Users frequently discussed the side effects of the medications (for e.g., diarrhoea, nausea, stomach upset, vomit, and digestive issues). There were also concerns about potential health risks associated with GLP-1 RAs (e.g., pancreatitis, kidney problems, and other adverse effects); tips and strategies for managing the side effects were shared between users.
- *Alcohol consumption and cravings*: users shared more detailed experiences regarding the reduced desire for alcohol, decreased cravings, and even quitting alcohol altogether whilst on these medications.
- *Positive and negative experiences*: users shared a range of experiences with the medications (positive experiences included benefits regarding alcoholic habits and weight loss; negative experiences were mainly about side effects and challenges in managing cravings and maintaining a healthy lifestyle).

Examples of relevant posts

- **"*Reduced*" reports:**

E.g., Has an added benefit of reducing your overall alcohol consumption. Before Wegovy I abused alcohol and just 3 weeks in my use is a fraction of what it used to be.
E.g., So, the oz has significantly reduced my cravings for alcohol, which is a good thing, but I also liked drinking and getting ""drunk"". (on weekends - not a nightly thing! but judge away, if you must) but my question is ----even when my stomach or body allows me to drink 5 or 6 beers - I am not even remotely feeling any of it. I used to have 1 draft of an IPA and I'd feel good...and now - nothing. So, it's pointless. Not sure if this happened to anyone else....
E.g., Yes! Now that I'm on a higher dosage I have almost zero interest in alcohol.
E.g., It's definitely impacted my alcohol cravings and I'm way less interested in anything more than a glass of wine which is so so nice.
E.g., I started MJ in Sept and now have moved to Wegovy. My alcohol desire has DRASTICALLY reduced.

- **"*Stopped*" reports:**

E.g., Within two weeks alcohol use and cravings started to disappear and I had been struggling with increased alcohol usage for almost a year, but not wanting to acknowledge it. Just started month 3 and I don't drink at all whereas I was drinking 4-5 days a week prior.
E.g., Same, 3 days in, no desire for cannabis or alcohol it's amazing
E.g., Lost all intrest for fast food and alcohol.
E.g., I'm 1.5 months in taking semaglutide (and 15.6 pounds down), and I just cannot believe how I don't crave alcohol anymore! I was the person who had a drink pretty much daily, my job entirely revolves around alcohol, and a post-shift drink (or 3) was a routine for me. I haven't bought any alcohol for my home, or cared

to go out and drink with my friends or coworkers at all! It is such an insane 180 from how I was before. This stuff is truly life changing
E.g., Alcohol cravings no longer exist. Before Wegovy, I would want a beer or glass of wine every day around 3pm. I'd think about it every 30 or 40 minutes until I had it. I would have 3 or 4 drinks a day. Everyday. Now? I don not think about when I may get to crack open a beer. (...). Its really very strange, but I'm so very very thankful!
E.g., I had a bit of a drinking problem before Ozempic. I haven't drank in the past year since Ozempic. Alcohol does nothing for me any more, no desire at all to drink.
E.g., And alcohol noise! Haven't wanted even a glass of wine in 8 months!
E.g., I have 0 interest in alcohol on Mounjaro…. Just social drinker, but complete lack of desire
E.g., everything tastes more and nausea. Also zero desire to even think of alcohol. carbs make me sick.
E.g., Yes , alcohol is now repulsive
E.g., The dark side is that I have no desire to drink alcohol and not able to attend happy hour 😩😩😩
E.g., I've been taking semaglutide (different medication, same drug class) for a few months and have lost 20% of my body weight and have stopped drinking alcohol or eating junk food. It's a miracle drug, and is covered by my insurance. It completely removes behaviors that are dopamine related- so eating junk food, eating rich foods, and even drinking alcohol and spending money. I saw a weight loss specialist, my BMI, pre- diabetes and history of dieting and exercising (many years) with little effect allowed me to qualify. I wish it had been around many years ago!
E.g., Makes sense. It makes your stomach feel heavy, so consuming ANYTHING becomes harder. So I would imagine it also makes consuming alcohol harder. I wasn't a huge alcoholic, but I would drink a beer maybe once a day. After getting on Mounjaro, I was always too full for beer, so I just stopped drinking.

- **"*Increased*" reports:**

E.g., I've had the complete opposite, it is heightened all my cravings for chocolate, alcohol and more food than I've ever eaten. I'm so pissed about that.
E.g., I know there has been a lot of discussions regarding alcohol drinking and ozempic. Mostly positive. I don't seem to be one of those and am seeking advice and compassion. No judgment please. I'm actually drinking more. It's like I need a drink or a few to feel happy and to sleep. I'm already beating myself up for this behavior. When I first started ozempic it was a miracle. I lost 20lbs and had no desire to drink alcohol or eat poorly. 1 year later things have changed. I keep telling myself tomorrow I'll be better but this past week especially I've had wine or beer every night. Am I alone? Does anyone else feel things are almost worst? I haven't gained the 20lbs back thankfully and am on .75mg ozempic weekly.

- **Other comments:**

E.g., Alcohol doesn't effect me now that I'm on Mounjaro, I can drink like a sailor and not feel anything.
E.g., Alcohol no longer affects me. I could drink a gallon and it's like water!
E.g., Interesting side effect my alcohol tolerance has gone way up. I'm normally a lightweight, but since increasing my dose to 5mg I just can't get drunk. I normally get pretty tipsy by 2 drinks, and drunk by 4 (I'm short), but now I'm barely tipsy at 4 drinks. I figure that the alcohol is leaving my stomach so slowly that I can't absorb it fast enough to become drunk. I also can't stomach more than 4 drinks otherwise I get too full. Also, I'm suuuuuper constipated. I'm drinking so much benefiber otherwise I get intense gas/constipation cramps. And my period and cramps have been all over the place. Nothing getting more water and fiber and NSAIDS can't fix, but interesting!
E.g., Time will also prove that this also works for some types of alcoholism.
E.g., I start craving alcohol. I don't think about/want/ desire alcohol at all the 5 days after my shot, but the 6th day hits and the craving is back. I began MJ for the weight loss, but I'm staying for the reduced food noise and cravings.
E.g., Friend took Wygovy & she had a decrease in her alcohol craving and lost weight. But cravings have resumed and wait is tiking back up.

E.g., I really think that this shot triggered changes in the habits in me that I had not noticed building up over years, habits that was using as crutches. Being able to "detox" off alcohol, anxiety and medication without feeling like the world was ending too? I think that resulted in getting pregnant.
E.g., My insurance won't cover my shots. I am paying out of pocket and it is breaking the bank (I drink for free- work in the industry so no savings there). I'm finding a way and will continue to do so as long as I can- having my alcoholism under control is priceless. It shouldn't be like that though- my insurance denying me a drug that is quite literally saving my life.
E.g., Yes. It's because Ozempic works by telling you're brain that everything is ok. There's no need get that instant hit of serotonin. People are reporting drinking less and it's being studied as a treatment for alcoholism. It's an interesting and hopeful drug in many ways.

- **Tirzepatide-related reports:**

E.g., As I've gotten used to tirz my alcohol craving has returned. Probably need to up my dose.
E.g., So i first started on ozempic and at first i felt an appetite suppression. After a while it went away. I got up to 1mg and still no change. I had to switch cause insurance would no longer cover it so I switched to tirzepatide. Im not at full dosage yet which I believe is 12-15. (...). I dont understand why i dont have the same effect. All ive done is maintain my weight. I do work in the alcohol industry so im constantly in bars n restaurants which i know is my problem but i was really hoping this would help with not wanting to drink or eat alot (...)I know this is not a miracle drug and you need to do the work. I just dont understand why it doesn't have the same effect on me that it did on my friends. I think about food constantly. Just curious if anyone is in the same boat.

Other drinking habits: coffee drinks. Keywords: caffeine; coffee

- *Medication impact on health and weight*: discussions about the impact of various medications on health, particularly related to thyroid-related issues, weight gain, and mental health.
- *Weight loss strategies and challenges*: numerous posts discussed different weight loss strategies, experiences with medications like Ozempic, and challenges faced during the weight loss journey.
- *Caffeine consumption and its effects*: the impact of caffeine on health, its role in weight loss strategies, and experiences of reducing or eliminating caffeine intake were discussed here. Some users reported a decreased desire for coffee and changes in taste preferences.
- *Metabolic and hormonal effects*: posts frequently mentioned metabolic changes, hormonal issues, and the challenges associated with maintaining or losing weight despite efforts.
- *Medication side effects and individual responses*: discussions about the side effects of various medications, individual responses to treatment, and efforts to manage or mitigate the impact of medication side effects were here discussed as well.

Examples of relevant posts

- **"*Reduced*" reports:**

E.g., I love coffee but I've noticed that I cannot finish a cup anymore and I usually drink 3 before work
E.g., I started about six weeks ago with all of the same symptoms. Everyone at been experiencing, however, a personal one that I have noticed is, I have leftover coffee! I really have not changed anything about my eating habits, but I definitely feel the heartburn and fullness, also less "food noise",.. but i always, ALWAYS have a medium iced coffee, less ice, 3cream 3caramel, no sugar. For the last three times that I've gotten coffee, I am unable to finish! Which is highly unusual for me! 🙌 its doing something yall!"
E.g., Has anyone else lost the coffee effect? Not only does it do nothing for me, anymore, I've also started to dislike the taste.
E.g., No online shopping, no alcohol, much reduced need for caffeine - almost zero anxiety and very clear thinking. At work, I am much more patient with my staff - it has really also helped with all of my menopause symptoms as I am not irritable or short-tempered at all.

- **"*Stopped*" reports:**

E.g., I'm 6 weeks in and I don't even think about coffee anymore.
E.g., I was addicted to coffee and now I don't even want to drink it!
E.g., Definitely also curbs interest in other enjoyable consumables, like (surprisingly) coffee. Never did drink much but have zero interest presently.
E.g., I've only taken my first 4 doses of 0.25mg… I almost immediately lost my desire to drink alcohol, coffee and smoke cigars… so I just stopped them… the only thing is, it didn't do anything for my blood sugar and appetite!!! Still eating like a pig… hoping it kicks in at 0.5mg in a few days!!! Also, dreams are crazy!!!!!!!!
E.g., My OCD, anxiety has improved, have zero interest/cravings for coffee, alcohol or sweets.

- **Other comments:**

E.g., I didn't have much in the way of side effects until I was put on max dosage of 4.5, the 2nd week of it. I've made a management plan and so far it's working. I highly recommend no caffeine and drinking a lot of ginger tea.
E.g., Coffee could be acidic, try a less acidic coffee (...).
E.g., drink coffee in the morning (no sugar obviously) it can help you to boost your metabolism and suppress the appetite, or drink tea .
E.g., Am I the only one that feels lonely? Food is a big thing in my culture, meal times and coffee breaks are sacred (...). Inviting someone to a meal is a way of bonding with them and I'm finding that most restaurants don't have menus or choices that I find appetizing or that I find won't upset my stomach. If I do order, I probably only eat 3 bites max and people also comment. (Not in a bad way, but they're concerned).

Tobacco. Keywords: nicotine; cigarette

- *Weight loss and related medications*: in this common theme, prevalent across multiple posts, users shared their experiences, their progress, and the impact of medications like Mounjaro and Ozempic on their weight loss journey.
- *Smoking cigarettes and substances*: discussions about cigarettes and other substances, as well as the potential impact of GLP-1 RAs on smoking, were identified here.
- *Abstinence and substance craving*: users posted personal stories of recovery from substances such as alcohol and discussed past struggles with cravings for alcohol and cigarettes.
- *Health and Risk Perception*: users discussed smoking health-related issues (e.g., smoking and cancer). Some posts highlighted the various risks associated with smoking.
- *Concerns about advertising and perceived inconsistencies in regulation*: Here, users expressed concerns about the perceived double standards in advertising regulations between cigarette companies and weight loss companies; while cigarette advertising is heavily restricted due to its known negative health effects, weight loss companies appear to face fewer constraints (e.g. about the impact on children's/young people's body image and eating behaviors), highlighting a perceived inconsistency in regulatory approaches between industries with potential public health implications.

Examples of relevant posts

- ***"Reduced"* reports:**

E.g., It's also helping with my cigarette cravings as well
E.g., I am definitely vaping less. And I have a vacation coming up in a few days and plan on using the change of scenery to quit entirely.
E.g., I still smoke, but way less. I'm still on the lower dosage (.5 ml), and a lot of testimonials say the addiction cravings start to wane when at the highest dose, but the alcohol has been the biggest for me. I also find certain foods I used to love unappealing, especially pastas, pizza, etc.. (...).

- ***"Stopped"* reports:**

E.g., Originally, I was just happy to have my A1C in the normal range and not to be craving food all the time but have lost only about 10 lbs. I was however able to finally quit my cigarette addiction for the last time. Ozempic made it so much easier to quit this time. I also stayed at 0.25 mgs. for a long time and have only been

at 50 mgs. for a short time - maybe it's time to bump it up to 1 mg. with my doctor's permission, of course. BTW do you try to stick within a certain calorie range and exercise regularly or just try to eat the right things when you feel like eating a little bit?
E.g., Idk, but I'm on week 6 of Ozempic and my carb and nicotine cravings are gone. Lost 16 pound so far.
E.g., SAME ! Took my first shot 12/19 and have not had a drink or cigarette since!!! 😮
E.g., I stopped smoking after the 3rd week on .5.
E.g., I quit sugar 3mths before I started Ozempic. That together with Ozempic developed a real sense of hope and I realised how deep down my powerlessness around my weight was weighing me down. That is a great feeing. But note it isn't a surrender and abstinance like when I quit drugs and alcohol or stopped smoking. Ozempic carries the hallmarks of a spiritually challenging drug.
E.g., I started semiglutide 2 months ago to help me loose a little weight. 3 days after my first injection I quit smoking. I have been a smoker for 12 years, and for about 11 years I've wanted to quit, but couldn't. (Wellbutrin wasn't enough to help me) Smoking was part of my daily routine and honestly, as superficial as this may sound, it helped me with my anxiety. I just woke up that 3rd day and felt absolutely no desire to smoke. Yesterday was 2 months no smoking!
E.g., Been a smoker for 20 plus years and been on Mounjauro since September of last year i have now I quit smoking.
E.g., I am diagnosed with binge eating disorder and Mounjaro had helped the mental portion of it. I also quit smoking as well

- **"*Started*" reports:**

E.g., Ughh I started smoking cigars when I started !

- **Other comments:**

E.g., Haven't had any side effects besides a weird pleasure feeling in my brain like I just smoked a cigarette (I quit years ago). This made me wonder if works like Chantix which has a side effect of disturbing thoughts.
E.g., What I don't understand is that we limit where cigarette companies advertise b/c of the affect their ads have on young children, but weight loss companies can advertise wherever despite the proven damage it does to children's body image and increased chance for them to develop disordered eating
E.g., I have tried so many times to quit smoking. no luck. I am told because of menthol. been on Ozempic for 8 months. 2mg. Help?
*E.g., This generation isn't far off smoking cr**k to loose weight , this shit is gonna affect you in the long run!!*
E.g., Works on the reward center, pt. actually requested for smoking cessation last week and we are trying semaglutide. I'll keep ya updated Failed chanti

---

Cannabis-derived products (smoked, inhaled, and edibles). Keywords: cannabis; weed; munchies; edibles

---

- *Cannabis use and impact on appetite*: users discussed the use of cannabis and its impact on appetite, late-night cravings, and nausea.
- *Medication and impact on eating habits*: users discussed the impact of medications like Ozempic on appetite, nausea, and weight loss.
- *Psychoactive substance use and mental health*: in this section, users posted some recommendations for using substances for managing mental health issues (anxiety, depression, PTSD).
- *Social and cultural perspectives*: discussions about various aspects (for e.g., societal views on ageing, beauty standards, and the role of substances in socializing) took place here.
- *Individual responses and experiences*: users discussed a range of individual responses to substances and medications and their impact on weight, cravings, and nausea.

Examples of relevant posts

- **"*Reduced*" reports:**

E.g., I smoke and consume a lot of weed. Since starting Wegovy, I noticed I've been partaking less. I also smoke for appetite suppression and energy but I guess I've been getting those from Wegovy so I might not be gravitating toward smoking as much? Wegovy has cut my tolerance a lot and I think it's a combo of the slow intestinal absorption (for edibles) and just smoking less.
E.g., Started on .25mg for 2 weeks and didn't really notice much of a change. A little bit of stomach upset and headaches. My hunger was reduced slightly, but nothing significant. I started on .5mg 4 days ago and now I see the impact. I can't eat more than half a meal now. Stomach is not feeling good at all. Food and water seems so unappealing. I force myself to have 100oz a day of water due to my predisposition to kidney stones. I stopped vaping completely. I have been smoking so much less pot. Not drinking coffee anymore even though I used to drink 3 cups every morning. The video game I play brings me no enjoyment anymore. Everything feels so boring and flat. It's really weird. Not sure what to make of it, but I am sure I will lose weight.
E.g., Has totally helped with my Amazon addiction and I have noticed a significant decrease in medical cannabis consumption
E.g., So, I will say that even though the munchies still exist, it's usually three cookies instead of 13. All in all, still a positive

- **"*Stopped*" reports:**

E.g., 1 week in and have had zero cannabis cravings a totally wild side effect I didn't realize would happen! Anyone else?
E.g., Same, 3 days in, no desire for cannabis or alcohol it's amazing

- **Other comments:**

E.g., Yeah Nah. I dry vape weed everyday do to the damn nausea as its the only thing that works and it hasn't in anyway changed my appetite in any way.
E.g., I smoked twice on it (used to be a heavy ass user). It subsided the nausea but I still didn't eat that much. Then again I used to use weed to curb my appetite so idk
E.g., Daily smoker - no munchies (on Ozempic). It does help with the nausea though!
E.g., I would so much rather smoke weed to deal with the nausea!
E.g., Even just a 10mg edible will make me want to eat everything in the fridge, even tho at that low a dose, I don't even really feel the high, or THC's anxiety-reducing effects, which is what I use it for. If I up the dose, resistance to food is futile. One of the best things about Ozempic for me is that it quiets the food noise in my brain. By which I mean that Ozempic frees up the mental and emotional energy I used to spend on either thinking about food or thinking about not eating food I very much want to scarf down. THC edibles erase that part of Ozempic's effects for me.
E.g., Gonna have to stop using pot tho cause it still gives me the munchies & next thing I know I've eaten a crap load of maltesers & as im trying to reduce my a1c, it's definitely not smart. I use pot for anxiety relief but have noticed the Wegovy takes away the anxiety, and it's improved my sleep, so hopefully my doc renews my script, cause I can totally see giving up pot.
E.g., I've had a mixed experience with edibles. Sometimes the munchies have been overpowering, sometimes they're really not at all. Either way, I'm not binging as much, because even with the munchies I eat significantly less. Edibles have hit me hard within a reasonable time frame and then some nights not at all, unless I slept through it, which is frustrating. I don't smoke or vape because I don't like feeling like my lungs are on fire, so I just use edibles and now I'm going to have to be more strategic with them, I guess.
E.g., (...) my husband and I live in a state where smoking marijuana is legal and it doesn't mess with my insulin levels so we gave up alcohol and exchanged it with pot. We don't smoke it because it's bad for your lungs. We take it in other ways. I know it's controversial But we feel pretty lucky that we can get it. You might find as your dosages go up that your system gets a little more sensitive.
E.g., Anyway, these days the medicinal aspect of it comes through when I'm not eating. it blows my mind in that weed is now what it's intended for . ok im kidding on intent but for me it has shifted from mostly recreational when i smoke to … i now smoke before i eat….. so i can eat.

Psychostimulants: cocaine and amphetamine-type stimulants. Keywords: cocaine; amphetamine

- *Comparison between prescription drugs and illicit substances*: users compared the effects of prescription weight loss drugs like Ozempic with illicit substances such as cocaine and methamphetamine. Discussions focussed on the perceived similarities in suppressing appetite.
- *Skepticism and negative views toward prescription drugs*: users expressed a range of concerns about safety, long-term side effects, and accusations of irresponsibility regarding Big Pharma promoting such medications without proper oversight.
- *Personal experiences with stimulants*: several users' posts involved personal experiences with different stimulants, including cocaine, amphetamine-based medications, and prescription drugs for ADHD. Users also discussed how these substances affect appetite, exercise, and overall well-being.
- *Concerns relating to the Pharma industry and weight loss approaches*: criticisms of the industry approach to weight loss were expressed in various posts. Some individuals suggested that doctors at times recommend drastic measures and prescribe drugs without adequate oversight. References to past practices, such as the promotion of amphetamine-based weight loss pills, were also made here.
- Humorous or sarcastic posts: some users expressed their opinions on weight loss methods, drug use, and the medical profession using humor. This theme appeared interesting for various reasons, because a range of individual coping strategies were commented.

Examples of relevant posts

E.g., I mean Cocaine has the same effect. It suppresses your hunger feeling so you are not hungry at all. Just saying for all you ""smart"" people out there who use this Ozempic, Crack also works, effects are 10x higher so you will properly lose even more weight, and IF YOU really want to lose weight fast and easy you can also just end your life, which i think is the best option. Its a WIN, WIN situation. You will lose weight!!! and the rest of use wont have to deal with your being so super "smart"

E.g., Had a bad habit with cocaine in 2016 for about a year. All good now, but the main reason I loved it? I never thought about food. All of that worry and planning and wanting to stuff myself? It was just gone. I didn't care about the high so much, but the freedom of not being constantly thinking about food.

E.g., You can get the same effect from some regular plain old methamphetamine or cocaine too. Pretty much any stimulant I've taken has suppressed the hell out of my appetite and increased the amount of exercise I got. I also forgot how to sleep and poop normally though.

E.g., diabetes drugs... which just happens to cause weight loss. Cocaine is excellent at weight loss too, doesn't mean everyone should be doing it. Was a t friends house this weekend when ozempic came up "I have some!" she declared & proceeded to show me all her injectors in the fridge. ""I had to jump through a bunch of hoops to get this, bought it from a spa I visit.."" (no prescription at all, just buying off an aesthetician she knows..). Most reports show that people go back to their "equilibrium weight" ("normal fatness") when they stop taking it & the long term side effects are not at all well known. Wildly irresponsible to push this crap.

E.g., Hmph. I'll stick with the amphetamine method, thanks. The tiny people that live in my walls swear by it.

Behavioral addictions (unrelated to food)

Compulsive shopping and other compulsive behaviors. Keywords: compulsive shopping; shopping

- *Medication experience*: discussions were made about the impact of GLP-1 RAs on weight loss, eating habits, and exercise routines; experiences with side effects (both positive and negative); positive outcomes and improvements in mental well-being.
- *Impact on food and eating behaviors*: the posts were related to a reduction in cravings and thoughts about food; detailed changes in eating habits (such as cutting out sweets and checking food for sugar content; freedom from constant thoughts about food).

- *Behavioral changes*: discussions were made about decrease in compulsive or impulsive behaviors (including shopping and alcohol consumption), changes in shopping habits (both in terms of frequency and spending), and a shift in focus from food-related activities to healthier choices.
- *Physical and mental health*: posts related to a positive impact on mental health, including reduced anxiety and clearer thinking; positive effects on overall well-being, leading to increased control over eating and life in general; and a concurrent reduction in the desire for substance consumption.
- *Challenges and concerns*: the range of discussions related to challenges with medications' side effects; concerns about the availability of medication in pharmacies, leading to difficulties in obtaining necessary supplies; and the exploration of alternatives and experiences with different medications.

Examples of relevant posts

- **"*Reduced*" reports:**

E.g., Yes! Less tv, less online shopping, less doom scrolling overall.
E.g., I have a really big shopping addiction, it is now greatly reduced.

- **"*Stopped*" reports:**

E.g., Your comments here feel like they came out of my own head. Spot on. I literally feel like my brain has freed up space it was using thinking about the next meal, grocery shopping, menus, and all the things. I give no crap about food right now. So when I eat I am making ridiculously healthy choices. This feels amazing. Good luck to you!!
E.g., My mental health has improved dramatically. I find myself feeling weird about feeling so normal and not hiding in my bedroom from the world. My binging or impulsive tendencies has ceased. Coffee, tea, Dr. Pepper and online shopping are not happening. I actually want to discuss lower doses or stopping some of my medications for my anxiety, depression and PTSD with my doctor. My normal has been just turn it off in my head and live in the shadows. I thought Serquel was a wonder drug because it gave me the mental health I needed but I gained 40 pounds. Now, that 40 plus more is gone and I drink water like wine.
E.g., 3 side effects I have had is not needing or wanting coffee in the morning! Not wanting or craving alcohol & the weirdest is No Desire to Shop!
E.g., Yessss….it's nearly impossible. I attribute it to the effect on the reward center of the brain chemistry being changed. It's also effected my desire to do anything that gives those hits of dopamine- shopping especially.
E.g., Yes!!! I stopped my impulsive amazon shopping! I've saved so much money. No internal battle. It's like my head is clear to make logical decisions.

- **"*Increased*" reports:**

E.g., I'm definitely drinking less. But I'm shopping more. 😂
E.g., Well I did post yesterday that I stopped biting my nails after 50 years but what I didn't say is I am shopping like a maniac.

- **Other comments:**

E.g., Two days after my first Ozempic shot, I felt it. All the constant food-related chatter that had been in my head for as long as I could remember was suddenly gone. It was like someone hit my mute button. I finally had the ability to choose foods that were healthy for my body. I had the ability to put down my fork when my stomach felt full. I had the ability to say ""no thanks"" when someone offered me a snack or a piece of candy. I had the ability to go grocery shopping without piling my cart full of candy and cookies and chips. I had the ability to leave the house without feeling the magnetic pull of the nearest drive-thru.
E.g., I've been on Mounjaro since January 23,2023. It's honestly worked great for me. I've lost 34lbs and I'm almost to goal. Gastrointestinal side effects have been constipation. The main other side effects that bother me the most… I don't feel like doing anything, ever! I'm tired, freezing, body aches, just blahhh. I don't drink, I can't eat, no joy in shopping anymore. I feel tired, little anxiety the first and second day after injection, and

bored lol 😂. I miss my old self without the extra weight! Anyone feel this way? Any suggestions on getting some energy and life back in me? I eat pretty decent, drink water, take a vitamin. Just laying on the couch under a blanket most of the time lol!

Sex drive and libido. Keywords: sex; libido

- *Weight loss and body image*: this theme was popular in individuals discussing significant weight loss experiences and the related impact on self-esteem and body image.
- *Sexual health and medication*: discussions about changes in libido, sexual experiences, and the impact of medications like Ozempic and Semaglutide on sex drive occurred here.
- *Relationship dynamics*: posts frequently related to relationship dynamics, including celibacy, concerns about partner reactions, and communication challenges affecting intimacy.
- *Health and wellness*: holistic health and connections between insulin control, hormones, and overall well-being were discussed here.
- *Medication use and side effects*: the use of medications, particularly semaglutide, and potential side effects were consistent themes.

Examples of relevant posts

- ***"Reduced"* reports:**

E.g., Noticed less drive but I'm okay with that as my libido now matches my SO.
E.g., Less alchol, less desire to spend and less sex.

- ***"Increased"* reports:**

E.g., (...) I've taken Ozempic for about a month and, to my surprise, sex drive has improved a lot. Does anyone else notice the same? I take antidepressants and they were relly affecting this aspect. I tried testosterone and gestrinone but none of them had too much effect
E.g., Hey girl heyy, yeah I started this month Feb 1, 2023. Yesterday I took my 4th .25 injection in my belly. Anyways, I have noticed too that my sex drive is coming back (...).
E.g., Recently our sex life has gone from good sex once a week to MINDBLOWING sex multiple times a week. Multiple orgasms , gasping for air, he feels bigger or maybe I feel tighter/ or both. He's "average" down below but I swear I feel like he's MASSIVE and I'm losing my virginity every time.
E.g., Any reports of increased sex drive? I've seen the opposite where people say sex drive was lower but for me…. The opposite, 44 and back to about 28 sex drive wise, just checking if this is normal. Strong like bull….
E.g., (...) Libido is through the roof, appetite is way down! (...).

The five most common themes overall

- *Medication effects and side effects*: this theme includes discussions about the effects of GLP-1RAs on habits, appetite, cravings, and weight loss. Potential side effects and health risks associated with these medications, such as digestive issues and long-term impacts on health, are also discussed here.
- *Lifestyle changes and weight management*: users discuss the importance of making lifestyle changes alongside medication intake for effective weight management (e.g., incorporating healthier dietary habits, engaging in physical activity, and other lifestyle modifications to support weight loss efforts).
- *Substance use and cravings reduction*: the users focus on the reduction in cravings for substances like alcohol, cigarettes, and sweets while on GLP-1RAs. Users also share their experiences with managing or quitting substance use.
- *Individual experiences and responses to GLP-1RAs*: this theme includes a range of individual experiences with medications (e.g., both positive and negative outcomes). Users share personal stories of weight loss progress, side effects, and overall responses to treatment, providing insights into the varied experiences with these medications.
- *Health risks, regulation, and societal perceptions*: discussions highlight users' concerns about health risks associated with medications and substance use, as well as societal perceptions

and regulatory inconsistencies (e.g., in the context of weight loss advertising and the pharmaceutical industry).
